# Identification of HIV-1 Epitopes that Induce the Synthesis of a R5 HIV-1 Suppression Factor by Human CD4^+^ T Cells Isolated from HIV-1 Immunized Hu-PBL SCID Mice

**DOI:** 10.1080/17402520500391557

**Published:** 2005-12

**Authors:** Atsushi Yoshida, Reiko Tanaka, Akira Kodama, Naoki Yamamoto, Aftab A. Ansari, Yuetsu Tanaka

**Affiliations:** 1 Department of Immunology Graduate School of Medicine University of the Ryukyus Uehara 207, Nishihara Okinawa 903-0215 Japan; 2 National AIDS Research Center National Institute of Infectious Diseases 1-23-1 Toyama, Shinjuku-ku Tokyo 162-8640 Japan; 3 Department of Microbiology and Immunology Emory University School of Medicine Atlanta GA 30322 USA

## Abstract

We have previously reported that immunization of the severe combined immunodeficiency (SCID) mice reconstituted with human peripheral blood mononuclear cells (PBMC) (hu-PBL-SCID mice) with inactivated human immunodeficiency virus type-1 (HIV-1)-pulsed-autologous dendritic cells (HIV-DC) elicits HIV-1-reactive CD4^+^ T cells that produce an as yet to be defined novel soluble factor *in vitro* with anti-viral properties against CCR5 tropic (R5) HIV-1 infection. These findings led us to perform studies designed to identify the lineage of the cell that synthesizes such a factor *in vitro* and define the epitopes of HIV-1 protein that have specificity for the induction of such anti-viral factor. Results of our studies show that this property is a function of CD4^+^ but not CD8^+^ T cells. Human CD4^+^ T cells were thus recovered from the HIV-DC-immunized hu-PBL-SCID mice and were re-stimulated *in vitro* by co-culture for 2 days with autologous adherent PBMC as antigen presenting cells, APC previously pulsed with inactivated HIV in IL-2-containing medium to expand HIV-1-reactive CD4^+^ T cells. Aliquots of these re-stimulated CD4^+^ T cells were then co-cultured with similar APC's that were previously pulsed with 10 μg/ml of a panel of HIV peptides for an additional 2 days, and their culture supernatants were examined for the production of both the R5 HIV-1 suppression factor and IFN-Υ. The data presented herein show that the HIV-1 primed CD4^+^ T cells produced the R5 suppression factor in response to a wide variety of HIV-1 gag, env, pol, nef or vif peptides, depending on the donor of the CD4^+^ T cells. Simultaneous production of human interferon (IFN)-Υ was observed in some cases. These results indicate that human CD4^+^ T cells in PBMC of HIV-1 naive donors have a wide variety of HIV-1 epitope-specific CD4^+^ T cell precursors that are capable of producing the R5 HIV-1 suppression factor upon DC-based vaccination with whole inactivated HIV-1.


*Clinical & Developmental Immunology*, December 2005; 12(4): 235–242

Taylor & Francis

Taylor & Francis Group

### Identification of HIV-1 epitopes that induce the synthesis of a R5 HIV-1 suppression factor by human CD4$^+$ T cells isolated from HIV-1 immunized hu-PBL SCID mice

ATSUSHI YOSHIDA[1], REIKO TANAKA[1], AKIRA KODAMA[1], NAOKI YAMAMOTO[2], AFTAB A. ANSARI[3], & YUETSU TANAKA[1]

[1]*Department of Immunology, Graduate School of Medicine, University of the Ryukyus, Uehara 207, Nishihara, Okinawa 903-0215, Japan,* [2]*National AIDS Research Center, National Institute of Infectious Diseases, 1-23-1 Toyama, Shinjuku-ku, Tokyo 162-8640, Japan, and* [3]*Department of Microbiology and Immunology, Emory University School of Medicine, Atlanta, GA 30322, USA*

**Abstract**

We have previously reported that immunization of the severe combined immunodeficiency (SCID) mice reconstituted with human peripheral blood mononuclear cells (PBMC) (hu-PBL-SCID mice) with inactivated human immunodeficiency virus type-1 (HIV-1)-pulsed-autologous dendritic cells (HIV-DC) elicits HIV-1-reactive CD4$^+$ T cells that produce an as yet to be defined novel soluble factor *in vitro* with anti-viral properties against CCR5 tropic (R5) HIV-1 infection. These findings led us to perform studies designed to identify the lineage of the cell that synthesizes such a factor *in vivo* and define the epitopes of HIV-1 protein that have specificity for the induction of such anti-viral factor. Results of our studies show that this property is a function of CD4$^+$ but not CD8$^+$ T cells. Human CD4$^+$ T cells were thus recovered from the HIV-DC-immunized hu-PBL-SCID mice and were re-stimulated *in vitro* by co-culture for 2 days with autologous adherent PBMC as antigen presenting cells, APC previously pulsed with inactivated HIV in IL-2-containing medium to expand HIV-1-reactive CD4$^+$ T cells. Aliquots of these re-stimulated CD4$^+$ T cells were then co-cultured with similar APC's that were previously pulsed with 10 μg/ml of a panel of HIV peptides for an additional 2 days, and their culture supernatants were examined for the production of both the R5 HIV-1 suppression factor and IFN-γ. The data presented herein show that the HIV-1 primed CD4$^+$ T cells produced the R5 suppression factor in response to a wide variety of HIV-1 gag, env, pol, nef or vif peptides, depending on the donor of the CD4$^+$ T cells. Simultaneous production of human interferon (IFN)-γ was observed in some cases. These results indicate that human CD4$^+$ T cells in PBMC of HIV-1 naive donors have a wide variety of HIV-1 epitope-specific CD4$^+$ T cell precursors that are capable of producing the R5 HIV-1 suppression factor upon DC-based vaccination with whole inactivated HIV-1.

**Keywords:** *HIV-1, vaccination, dendritic cells, HIV-1 suppression*

**Abbreviations:** *HIV-1, human immunodeficiency virus type I; DC, dendric cells; HIV-DC, inactivated HIV-1-pulsed DC; Th, helper T; hu-PBL-SCID mouse, severe combined immunodeficiency mouse engrafted with human PBMC; AT-2, aldrithiol-2; 50% TCID$_{50}$, tissue culture infectious dose*

### Introduction

Virus specific CD4$^+$ helper T (Th) cell responses have been shown to play an essential role in the maintenance of effective immune responses in a variety of animal models [3,12,20,32,34]. Human immunodeficiency virus type 1 (HIV-1) infection is associated with a progressive loss of total CD4$^+$ Th cells by both direct and indirect mechanisms [21,25,27,28,30]. In particular, memory CD4$^+$ Th cells become more susceptible to the cytopathic effects of HIV-1 than naive CD4$^+$ Th cells after activation [8]. Several lines of evidence strongly suggest that HIV-1-specific CD4$^+$ Th cells are critical for control

Correspondance: Dr Y. Tanaka, Department of Immunology, Graduate School of Medicine, University of the Ryukyus, Uehara 207, Nishihara-cho, Nakagami-gun, Okinawa 903-0215, Japan. Tel: 81 98 895 1202. Fax: 81 98 895 1437. E-mail: yuetsu@ma.kcom.ne.jp

ISSN 1740-2522 print/ISSN 1740-2530 online © 2005 Taylor & Francis
DOI: 10.1080/17402520500391557

of HIV-1 in part by maintaining HIV-1-specific $CD8^+$ cytotoxic T lymphocyte (CTL) responses [13,17,22,23,26,28]. Highlighting the complexity of HIV-1 infection was the finding that HIV-1-specific $CD4^+$ Th cells were in fact preferentially infected by HIV-1 *in vivo* [9]. It was reasoned that such mechanisms may indeed contribute to an impairment in the control of not only HIV-1 but also opportunistic infections. While such $CD4^+$T cell depletion continues to occur with variable kinetics in HIV-1 infected patients, a significant frequency of HIV-1-specific $CD4^+$ Th cells are detectable in most of these individuals with a higher frequency in long-term non-progressors (LTNP) than in subjects during progressive disease [4,28,37,38]. In addition to the traditional function of $CD4^+$ T cells in facilitating the generation of HIV-1 specific $CD8^+$ T cells and the synthesis of antibodies, $CD4^+$ Th cells from HIV-1 infected patients have also been shown to exert anti-viral effects not only by direct lysis of HIV-1 infected target cells by HIV-1 gag specific $CD4^+$ T cells [18] but also by the secretion of a variety of HIV-1 suppression factors [1,15,31,41]. These findings together suggest that $CD4^+$ T cells from HIV-1 infected and/or immunized individuals have acquired a series of unique anti-viral activity which may contribute to the control of viremia and the detailed studies of the mechanisms by which such activity is acquired and induced thus appears warranted. We have previously reported that SCID mice engrafted with HIV-1-naive human PBMC together with autologous DC pulsed with inactivated whole HIV-1 virion became subsequently completely resistant to R5 HIV-1 challenge *in vivo*. The resistance to infection was specific since it was only seen in the hu-PBL-SCID mice immunized with inactivated R5 or X4 HIV-1 virions, but not OVA or KLH. Studies of the mechanisms by which these mice were protected led to the discovery of a novel soluble anti-viral factor produced in the serum of these HIV-DC immune mice. While this factor synthesized by human $CD4^+$ T cells from the hu-PBL-SCID HIV-1 immunized mice was unable to neutralize HIV-1 *in vitro*, it was capable of inhibiting R5, but not X4 HIV-1 infection of primary macrophages and activated PBMC which was not secondary to the down regulation of either CCR5 or CD4 and appeared to act prior to viral integration. Of interest was also the finding that the factor was not effective in controlling R5 HIV-1 infection of CCR5-expressing $CD4^+$T cell lines.

Since the generation of the suppressor factor appeared to be specifically induced by HIV-1 immunization of the hu-PBL-SCID mice, it was reasoned that the delineation of the epitopes of the HIV-1 encoded proteins that induce the generation of such factors would be appropriate and informative. Results of such studies are the basis of this report.

### Materials and methods

### *Mice*

SCID mice lacking functional T, B and natural killer cells (NK), BALB/c-rag2$^{-/-}$ $\gamma c^{-/-}$ [24] were used in the present study. The mice were kept in a SPF and BSL-3 animal facilities of the Laboratory Animal Center, University of the Ryukyus. The protocols for the care and use of hu-PBL-SCID mice have been approved by the committee on animal research of the University of the Ryukyus.

### *Reagents*

RPMI 1640 medium (SIGMA, St Louis, MO) supplemented with 5% fetal calf serum (FCS), 100 U/ml of penicillin and 100 μg/ml of streptomycin (hereinafter called RPMI medium), serum free medium (AIM-V) and Iscove's medium (Lifetechnology, NY) supplemented with 10% FCS with the antibiotics (hereinafter called AIM-V medium and Iscove's medium) were utilized as sources of media. Soluble recombinant human IL-4 and GM-CSF were produced in 293 T cells transfected with pCMhIL4 and pCMhGM (RIKEN Gene Bank, Ibaraki, Japan), respectively, by the calcium phosphate method [33] and purified. Concentrations of human IL-4 and GM-CSF were determined using commercial ELISA kits (BioSource, Camarillo, CA). Human recombinant IL-2 was kindly supplied by NIH AIDS Research and Reference Program.

Blocking monoclonal antibodies (mAb) against human MIP-1α, human MIP-1β and human RANTES were purchased from R&D systems (Rockville, MD). To maintain their blocking activity, these antibodies in a lyophilized form were reconstituted in accordance with the manufacturer's instructions, and aliquots were kept at −80°C until use. Heparin–Sepharose (Pharmacia, Sweden) was used to absorb β-chemokines as described previously in Ref. [39].

HIV-1 peptides were obtained from the NIH AIDS Research and Reference Program, including the complete sets of the HIV-1 HXB2 gag peptides (Cat#5107 and Cat#3992), HIV-1 MN env peptides (Cat#6451), HIV-1 HXB2R pol peptides (Cat#4358), HIV-1 clade B nef peptides (Cat#5189), HIV-1 consensus B vif peptides (Cat#6445), HIV-1 consensus B rev peptides (Cat#6445), HIV-1 BRV nef peptides (Cat#6441) and HIV-1 consensus vpu peptides (Cat#6444). Each peptide was dissolved in DMSO at a concentration of 10 mg/ml, and then diluted in RPMI medium at 10 μg/ml prior to use.

### *Viruses*

HIV-$1_{JR\text{-}CSF}$ [16] and HIV-$1_{NL4\text{-}3}$ [2] viral stocks were each produced in the 293T cells by transfection with the appropriate HIV-1 infectious plasmid DNA utilizing the calcium phosphate method [33]. HIV-$1_{IIIB}$ was harvested from Molt-4/IIIB cell cultures. The 50% tissue culture infectious dose ($TCID_{50}$) was determined by an endpoint infectious assay using PBMC stimulated with anti-CD3/anti-CD28-mAb conjugated DynaBeads (Dynal, Oslo, Norway). HIV-1 preparations were inactivated with Aldrithiol-2 (AT-2), as originally described by Rossio et al. [29].

*Generation of HIV-1 pulsed mature DC's from monocytes*

Fresh PBMC at $5 \times 10^6$ cells/ml in RPMI medium were dispensed into individual wells of 12-well plates (1 ml/well), which had been precoated with autologous plasma for 30 min at 37°C. The PBMC cultures were allowed to incubate at 37°C for 1 h. The non-adherent cells were removed by gentle washing with serum-free RPMI 1640 medium and the remaining adherent cells were cultured in Iscove's medium (2 ml/well) containing human GM-CSF (500 ng/ml) and IL-4 (200 ng/ml) for 5 days. The resulting immature DC cultures were depleted of contaminating lymphocytes by using the monocyte-negative isolation kit (Dynal, Oslo, Norway), and the enriched population of DC's were further cultured at $2 \times 10^6$/ml in human IFN-β (1000 U/ml; Toray, Tokyo, Japan) in the presence of AT-2 inactivated whole HIV-1 (containing 50 ng/ml p24) for 2 days to obtain mature DC pulsed with HIV-1, as described previously in ref. [39].

*Transplantation and immunization*

HIV-1-pulsed mature DC ($5 \times 10^5$ cells) mixed with autologous fresh PBMC ($3 \times 10^6$ cells), or those depletes of $CD8^+$ T cells, or $CD4^+$ T cells by a magnetic isolation method (Dynal, Oslo, Norway) in a final volume of 100 μl in RPMI medium were injected into the spleen of SCID mice. Five days later, the same number of DC pulsed with the same dose of HIV-1 antigen were inoculated into the peritoneal cavity. Five days later, the mice were sacrificed and human lymphocytes were recovered from the spleen and the peritoneal cavity by lavage.

In vitro *re-stimulation of $CD4^+$ T cells*

The recovered human lymphocytes from the HIV-DC immune mice were depleted of human $CD8^+$ cells by the magnetic beads-negative selection method (Dynal). These enriched population of human $CD4^+$ T cells ($2 \times 10^6$ cells) were co-cultured with freshly obtained $2 \times 10^5$ autologous mitomycin-C treated adherent PBMC as antigen presenting cells (APC), in the presence of AT-2 inactivated HIV-1 containing 50 ng p24 in a volume of 1 ml RPMI 1640 medium supplemented with 20 U/ml human IL-2 in individual wells of a 24-well plate (BD Pharmingen, San Diego, CA) at 37°C. Two days later, these $CD4^+$ T cells were assayed for HIV-1 peptide specific responses as follows. An initial screening was performed utilizing pools of 10 sequential overlapping peptides and once a pool of peptides was shown to lead to the synthesis of the HIV-1 suppression factor, a second screening was performed to identify the individual peptide. Thus, enriched population of $2 \times 10^4$ autologous APC were first dispensed in a volume of 50 μl into individual wells of a 96-well microtiter plate and triplicate wells incubated with 50 μl of media containing the various individual pools of 10 HIV-1 peptides for the primary screen (each at 10 μg/ml) or an individual HIV-1 peptide for the secondary screen (10 μg/m) for 1 h at 37°C. A total of $2 \times 10^5$ HIV-1 primed and *in vitro* re-stimulated $CD4^+$ T cells were then added to these cultures in a volume of 100 μl of media and the co-cultures incubated at 37°C in a 5% $CO_2$ humidified atmosphere. Two days later, the culture supernatants were harvested and aliquots assayed for HIV-1 inhibition activity and levels of IFN-γ.

*HIV-1 inhibition and IFN-γ assays*

As previously reported [39], PBMC were activated with anti-CD3/28 antibody-coated magnetic beads (Dynal) at cell to bead ratio of 1:1 in 20 U/ml IL-2 containing RPMI medium *in vitro* for 3 days. These activated PBMC's ($5 \times 10^5$ cells/well) were pre-incubated at 37°C for 1 h with either various dilution of serum from the HIV-DC immune mice or culture supernatants obtained from the $CD4^+$ T cells that were stimulated with various HIV-1 peptides as described above. Five hundred $TCID_{50}$ of HIV-$1_{JR\text{-}CSF}$ or HIV-1 $_{NL4\text{-}3}$ was then added to these cultures for an additional 4 h. The microtiter plate was centrifuged and the supernatant fluid removed. The procedure repeated three times and the cultures then were incubated in 0.2 ml of 20 U/ml IL-2-containing RPMI medium for 5 days. The levels of HIV-1 p24 produced in the culture supernatants were determined by a commercial ELISA kit (Zepto Metrix, Buffalo, NY). Levels of human IFN-γ in the stimulated culture supernatants were assayed utilizing a commercial ELISA kit (R&D Systems Inc., Minneapolis, MN).

*HLA typing*

Donor PBMC were HLA typed by a DNA typing method.

### Results

In order to identify the lineage of cells that was the major producer of the R5 HIV-1 suppression factor *in vivo*, SCID mice were engrafted with unfractionated

PBMC or PBMC depleted of either $CD4^+$ or $CD8^+$ cells, together with HIV-DC. Various dilutions of the immune sera from these mice were then examined for the R5 HIV-1 suppression activity *in vitro*. As seen in Figure 1, as little as 5% of the sera from the mice engrafted with either unfractionated PBMC or CD8-depleted PBMC significantly ($p < 0.05$) inhibited R5 HIV-1 infection. In contrast, up to 20% of the sera from the CD4-depleted PBMC-engrafted mice did not show any detectable R5 HIV-1 suppressive activity. Similar assays conducted using X4 HIV-1 failed to show any inhibition denoting that the inhibition was selective for R5 HIV-1 (data not shown). In these engrafted mice, the levels of human $CD4^+$ and $CD8^+$ T cells existed at similar levels as in the mice engrafted with unfractionated PBMC (data not shown). These data indicate that $CD8^+$ T cells are not required for the production of the R5 HIV-1 suppression factor *in vivo*, and that the HIV-immune human $CD4^+$T cell population is the major source for the R5 HIV-1 suppression factor *in vivo*.

In efforts to map the epitopes of HIV-1 antigens that lead to the activation of $CD4^+$ T cells and the subsequent release of the HIV-1 suppression factor, a large panel of 15–20 mer peptides spanning a wide range of HIV-1 proteins were screened using a re-stimulation assay as described in the methods section. In order to enrich for HIV-1 antigen-responding $CD4^+$ T cells *in vitro*, $CD8^+$ T cell-depleted lymphocytes obtained from three groups of HIV-DC immune hu-PBL-SCID mice, which had been engrafted with PBMC's from different individuals, were first stimulated with inactivated whole HIV-1 in the presence of autologous APC *in vitro*. These HIV-1-primed enriched $CD4^+$ T cells were then re-stimulated by co-culture in the presence of a pool of 10 HIV-1 peptides with fresh APC for primary screening. After 2 days, culture supernatants were harvested and examined for the R5 HIV-1 suppression factor at a final dilution of 50%. Supernatant fluids giving values of >50% reduction compared to controls (incubated with media alone) in p24 antigen synthesized in the R5 HIV-1 infected cells were considered positive and a secondary screen performed to identify the individual HIV peptide inducing such HIV-1 suppression factor activity. In order to make sure that the R5 HIV-1 suppression was not mediated by CCR5-binding β-chemokines, the culture supernatants to be tested were individually pre-absorbed by incubation with Heparin–Sepharose followed by incubation with a pool of blocking mAbs against human RANTES, MIP-1α and MIP-1β each a concentration of 10 μg/ml. Figure 2a–c shows representative data from a number of independent experiments conducted on cells from the three donors, respectively. These data suggest that the ability of HIV-1 peptides to induce the HIV-1 suppressor factor resides in multiple HIV-1 peptides from a number of HIV-1 proteins. As expected, there were variations noted in the HIV-1 proteins and peptides that induce the HIV-1 suppression factor in the three individual donors. The peptides recognized by donor 1 $CD4^+$ T cells consisted of peptides from HIV-1 pol, gag and env antigens, while those recognized by donor 2 were of gag, env, vif and rev antigens, and those recognized by donor 3 were of gag, env, vif and rev antigens. None of the supernatant fluids examined showed detectable X4 HIV-1 suppression activity in the assay employed (data not shown). There was specificity in the assay since the R5 HIV-1 suppression factor was not detected either in the culture supernatants from APC cultured with or without HIV-1 peptides alone or supernatant fluids from immune $CD4^+$ T cells cultured in the absence of APC or peptides (data not shown). The individual variations in the response to peptides may be due to HLA restriction of responding antigenic peptides, as the three donors were of different HLA class II type (Table I). It is interesting to note that the production of the R5 HIV-1 suppression factor by the bulk $CD4^+$ T cells was

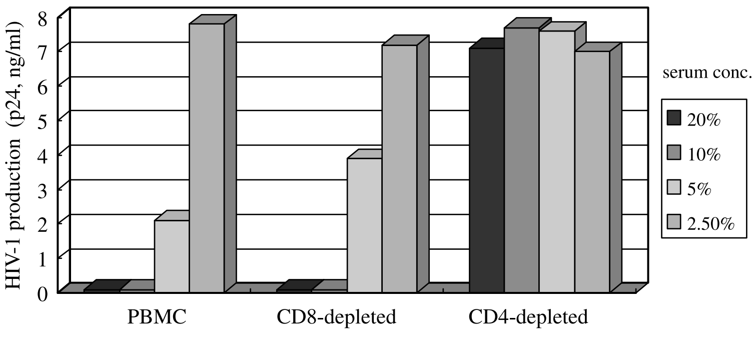

Figure 1. Requirement of human $CD4^+$ T cells, but not $CD8^+$ T cells, to generate the R5 HIV-1 suppression factor in the DC-HIV-immune hu-PBL-SCID mice. Together with HIV-DC, PBMC or those depleted of $CD8^+$ or $CD4^+$ cells were engrafted into the spleen of four SCID mice per group. After 5 days, mice were boosted with HIV-DC. After 5 days, serum samples from each group were pooled and tested for the R5 HIV-1 suppression activity on activated PBMC. Bars show the means of HIV-1 p24 values in the PBMC culture supernatants on day 5 post infection. HIV-1 proliferation in medium control was 7.7 ng/ml.

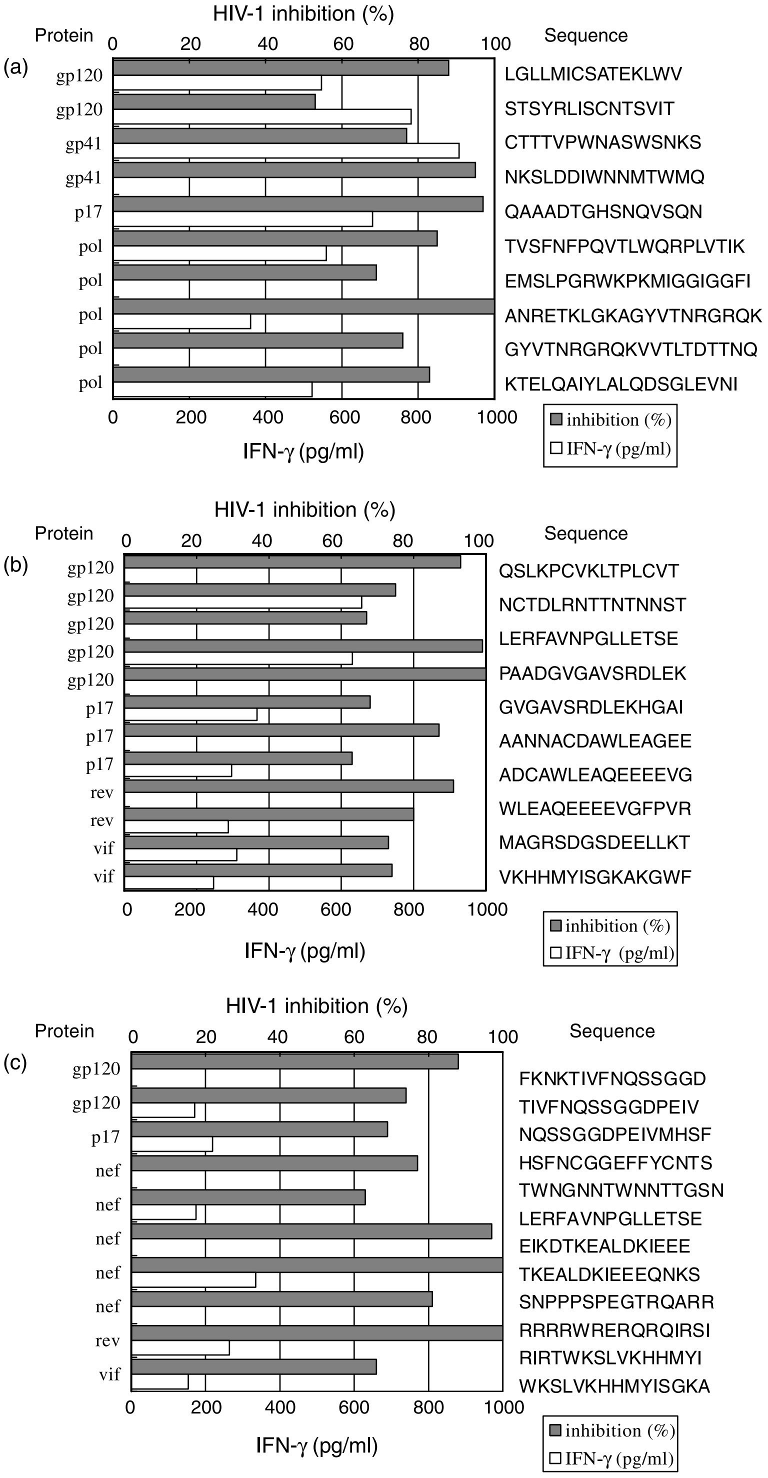

Figure 2. Recognition of various HIV-1 peptides by HIV-1-immune human $CD4^+$ T cells from three different donors generated in the HIV-DC-immune hu-PBL-SCID mice. HIV-DC immune $CD4^+$ T cells ($2 \times 10^5$ cells) were stimulated with various peptides at a concentration of 10 μg/ml in the presence of $2 \times 10^4$ APC in a volume of 200 μl in individual wells of a 96-well plate. After 2 days, the culture supernatants were harvested and their activity of R5 HIV-1 inhibition and the levels of human IFN-γ were determined as described in the materials and methods. Percent inhibition of HIV-1 production was calculated using values obtained from HIV-1 infected PBMC that were pre-treated with the medium alone. Representative data from three independent experiments for donor 1 (a), donor 2 (b), and donor 3 (c) were shown.

Table I. HLA class II typing.

| | DR | DQ |
|---|---|---|
| Donar 1 | 15/– | 6/– |
| Donar 2 | 4/14 | 4/7 |
| Donar 3 | 4/13 | 6/8 |

accompanied by the production of human IFN-γ but not in all the cases.

### Discussion

Our laboratory has previously documented the synthesis of a soluble factor by CD4$^+$ T cells isolated from SCID mice previously engrafted with a mixture of human PBMC's and HIV-1-pulsed autologous DC's which suppressed infection of R5 HIV-1 *in vitro* [39]. In the present study we have identified the CD4$^+$T cell lineage as a requirement for the synthesis of this factor *in vivo*. In addition, we have attempted to identify the HIV-1 epitopes that are recognized by CD4$^+$ T cells that lead to the synthesis of this factor. There are a number of issues that need to be addressed in light of these findings. Thus, first of all, we submit that the anti-viral factor is distinct from the other anti-viral factors that have been previously identified. The fact that the suppressor activity was not neutralized by the addition of antibodies against the chemokines RANTES, MIP-1α and MIP-1β suggest that such suppression is not likely due to the synthesis of these chemokines which are known inhibitors of viral infection [7,36]. The factor described herein is also distinct from the cellular anti-viral factor (CAF) identified by the laboratory of Dr Jay Levy [19] since it is synthesized by CD4$^+$ T cells but not CD8$^+$ T cells and whereas Dr Levy's CAF is effective against all HIV-1, HIV-2 and select retroviruses. The factor reported herein appears to be specific for R5 but not X4 tropic HIV-1. In addition, it is not likely to be either lymphotactin [35], α-defensins [40], the heparin binding protein termed anti-thrombin III [10], the natural killer enhancing factors A and B [11], and some additional factors (reviewed by Levy et al. [5]) since all of these have been shown to be synthesized primarily by CD8$^+$ but not CD4$^+$ T cells. There has been an anti-viral factor that is synthesized following the interaction of CD4$^+$ T cells with APC's [6]. However, this factor is functionally distinct from our factor because the factor inhibits HIV-1 production at post-integration stage, and our factor inhibits R5 HIV-1 infection prior to integration [39]. It is clear though that there are a plethora of such anti-viral factors that have been and continue to be described. The biologic reasons for such a multitude of anti-viral factors is difficult to imagine except to state that these are likely due to redundancies that nature has bestowed on the vertebrate species to protest itself from such viral infections.

The second issue concerns the multiple epitopes of HIV-1 that induce the generation of this factor by CD4$^+$ T cells. These epitopes interestingly were not restricted to a single HIV-1 protein and included both viral structural and accessory proteins. The obvious individual variations in responding antigenic peptides suggests that this maybe due to HLA-restriction of peptide recognition by the immune CD4$^+$ T cells, although additional studies are necessary to confirm this issue. Furthermore, the present study suggests that the CD4 factor-producing HIV-1-immune CD4$^+$ T cells are heterogeneous Th clones. Since the peptides tested in this study were overlapping 15–20 mers, an additional study is required to more precisely define the specific HIV-1 epitope that has specificity for the CD4$^+$ T cells. It is also unclear whether the suppressor factor synthesized by CD4$^+$ T cells in response to distinct HIV peptides is identical or distinct. Biochemical identification of the factor(s) is needed to address this issue and such studies are in progress.

The next issue concerns the synthesis of IFN-γ by some but not all the CD4$^+$T cell in the assays reported herein. Since the cultures performed consisted of a mixture of CD4$^+$ T cells, it is not clear whether the synthesis of the anti-viral factor and IFN-γ was the result of a single clonal population of multiple clones of CD4$^+$ T cells. Clonal analysis needs to be performed to address this issue.

It is important to note that the HIV-1 gag p17 antigen peptide (*LERFAVNPGLLETSE*) recognized by the HIV-DC immune CD4$^+$ T cells from two out of the three donors shares amino acid sequence with another gag p17 peptide (*ERFAVNPGLLETSEGCR*) that is widely recognized by IFN-γ-producible CD4$^+$ T cells from at least 25% HIV-1-infected individuals [14]. These results strongly support the use of DC-based vaccination of hu-PBL-SCID mice with whole inactivated HIV-1 virion to stimulate and expand HIV-1-specific CD4$^+$ T cells in efforts to study the effectiveness of these cells for anti-viral control. More importantly, it should be noted that the CD4 factor producing CD4$^+$ T cells generated in the hu-PBL-SCID mice recognize multiple HIV-1 proteins similar the studies that have reported for HIV-1-specific IFN-γ producing CD4$^+$ T cells in HIV-1 infected individuals [39]. Although, the existence and clinical role of the CD4 factor in HIV-1-infected individuals remains unknown, the data reported herein suggest that HIV-1 vaccines containing multiple HIV-1 epitopes or proteins, rather than those with single HIV-1 protein or epitope, will be likely to be more effective in expanding a large number of multiple HIV-1-reactive CD4$^+$ T cells that are capable of producing the CD4 factor and other helper and HIV-1-suppresing cytokines.

As far as we know up to present, there have been no reports on cytokines that are identical to the CD4 anti-viral factor described herein. Attempts to identify the biochemical nature of this novel anti-viral factor have been hampered by both our inability to prepare sufficiently large quantities that are required for both biochemical identification and detailed characterization of this molecule coupled with the labile nature of the molecule. Fortunately, in our recent attempts, we have succeeded in immortalizing CD4 factor-producing $CD4^+$ T cells by HTLV-I-mediated transformation (Yoshida et al. unpublished). These cells will be helpful for the identification of not only the factor itself, but also its putative receptor.

### Acknowledgements

This work was supported by grants from a Grant-in-Aid for Scientific Research on Priority Areas from the Ministry of Education, Culture, Sports, Science, and Technology of Japan; Research on HIV/AIDS and Health Sciences focusing on Drug Innovation from the Ministry of Health, Labor and Welfare of Japan; and Japan Human Science Foundation.

### References

[1] Abdelwahab SF, Cocchi F, Bagley KC, Kamin-Lewis R, Gallo RC, DeVico A, Lewis GK. 2003. HIV-1-suppressive factors are secreted by $CD4^+$ T cells during primary immune responses. Proc Natl Acad Sci USA 100:15006–15010, Epub 2003 Dec 1.

[2] Adachi A, Gendelman HE, Koenig S, Folks T, Willey R, Rabson A, Martin MA. 1986. Production of acquired immunodeficiency syndrome-associated retrovirus in human and nonhuman cells transfected with an infectious molecular clone. J Virol 59:284–291.

[3] Battegay M, Moskophidis D, Rahemtulla A, Hengartner H, Mak TW, Zinkernagel RM. 1994. Enhanced establishment of a virus carrier state in adult $CD4^+$ T-cell-deficient mice. J Virol 68:4700–4704.

[4] Betts MR, Ambrozak DR, Douek DC, Bonhoeffer S, Brenchley JM, Casazza JP, Koup RA, Picker LJ. 2001. Analysis of total human immunodeficiency virus (HIV)-specific CD4(+) and CD8(+) T-cell responses: Relationship to viral load in untreated HIV infection. J Virol 75:11983–11991.

[5] Blackbourn DJ, Mackewicz C, Barker E, Levy JA. 1994. Human $CD8^+$ cell non-cytolytic anti-HIV activity mediated by a novel cytokine. Res Immunol 145:653–658.

[6] Butera ST, Pisell TL, Limpakarnjanarat K, Young NL, Hodge TW, Mastro TD, Folks TM. 2001. Production of a novel viral suppressive activity associated with resistance to infection among female sex workers exposed to HIV type 1. AIDS Res Hum Retroviruses 17:735–744.

[7] Capobianchi MR, Abbate I, Antonelli G, Turriziani O, Dolei A, Dianzani F. 1998. Inhibition of HIV type 1 BaL replication by MIP-1$\alpha$, MIP-1$\beta$, and RANTES in macrophages. AIDS Res Hum Retroviruses 14:233–240.

[8] Chun TW, Chadwick K, Margolick J, Siliciano RF. 1997. Differential susceptibility of naive and memory $CD4^+$ T cells to the cytopathic effects of infection with human immunodeficiency virus type 1 strain LAI. J Virol 71:4436–4444.

[9] Douek DC, Brenchley JM, Betts MR, Ambrozak DR, Hill BJ, Okamoto Y, Casazza JP, Kuruppu J, Kunstman K, Wolinsky S, Grossman Z, Dybul M, Oxenius A, Price DA, Connors M, Koup RA. 2002. HIV preferentially infects HIV-specific $CD4^+$ T cells. Nature 417:95–98.

[10] Geiben-Lynn R, Brown N, Walker BD, Luster AD. 2002. Purification of a modified form of bovine antithrombin III as an HIV-1 $CD8^+$T-cell antiviral factor. J Biol Chem 277:42352–42357.

[11] Geiben-Lynn R, Kursar M, Brown NV, Addo MM, Shau H, Lieberman J, Luster AD, Walker BD. 2003. HIV-1 antiviral activity of recombinant natural killer cell enhancing factors, NKEF-A and NKEF-B, members of the peroxiredoxin family. J Biol Chem 278:1569–1574.

[12] Janssen EM, Lemmens EE, Wolfe T, Christen U, von Herrath MG, Schoenberger SP. 2003. $CD4^+$ T cells are required for secondary expansion and memory in CD8+ T lymphocytes. Nature 421:852–856.

[13] Kalams SA, Buchbinder SP, Rosenberg ES, Billingsley JM, Colbert DS, Jones NG, Shea AK, Trocha AK, Walker BD. 1999. Association between virus-specific cytotoxic T-lymphocyte and helper responses in human immunodeficiency virus type 1 infection. J Virol 73:6715–6720.

[14] Kaufmann DE, Bailey PM, Sidney J, Wagner B, Norris PJ, Johnston MN, Cosimi LA, Addo MM, Lichterfeld M, Altfeld M, Frahm N, Brander C, Sette A, Walker BD, Rosenberg ES. 2004. Comprehensive analysis of human immunodeficiency virus type 1-specific CD4 responses reveals marked immunodominance of gag and nef and the presence of broadly recognized peptides. J Virol 78:4463–4477.

[15] Kinter AL, Ostrowski M, Goletti D, Oliva A, Weissman D, Gantt K, Hardy E, Jackson R, Ehler L, Fauci AS. 1996. HIV replication in CD4+ T cells of HIV-infected individuals is regulated by a balance between the viral suppressive effects of endogenous beta-chemokines and the viral inductive effects of other endogenous cytokines. Proc Natl Acad Sci USA 93:14076–14081.

[16] Koyanagi Y, Miles S, Mitsuyasu RT, Merrill JE, Vinters HV, Chen IS. 1987. Dual infection of the central nervous system by AIDS viruses with distinct cellular tropisms. Science 236:819–822.

[17] Krowka JF, Stites DP, Jain S, Steimer KS, George-Nascimento C, Gyenes A, Barr PJ, Hollander H, Moss AR, Homsy JM, et al. 1989. Lymphocyte proliferative responses to human immunodeficiency virus antigens *in vitro*. J Clin Invest 83:1198–1203.

[18] Lotti B, Wendland T, Furrer H, Yawalkar N, von Greyerz S, Schnyder K, Brandes M, Vernazza P, Wagner R, Nguyen T, Rosenberg E, Pichler WJ, Brander C. 2002. Cytotoxic HIV-1 p55gag-specific $CD4^+$ T cells produce HIV-inhibitory cytokines and chemokines. J Clin Immunol 22:253–262.

[19] Mackewicz CE, Craik CS, Levy JA. 2003. The CD8+ cell noncytotoxic anti-HIV response can be blocked by protease inhibitors. Proc Natl Acad Sci USA 100:3433–3438.

[20] Matloubian M, Concepcion RJ, Ahmed R. 1994. $CD4^+$ T cells are required to sustain CD8+ cytotoxic T-cell responses during chronic viral infection. J Virol 68:8056–8063.

[21] McCune JM. 2001. The dynamics of $CD4^+$ T-cell depletion in HIV disease. Nature 410:974–979.

[22] McMichael AJ, Rowland-Jones SL. 2001. Cellular immune responses to HIV. Nature 410:980–987.

[23] Migueles SA, Laborico CA, Shupert WL, Sabbaghian MS, Rabin R, Hallahan CW, Van Baarle D, Kostense S, Miedema F, McLaughlin M, Ehler L, Metcalf J, Liu S, Connors M. 2002. HIV-specific $CD8^+$ T cell proliferation is coupled to perforin expression and is maintained in nonprogressors. Nat Immunol 3:1061–1068.

[24] Ohteki T, Fukao T, Suzue K, Maki C, Ito M, Nakamura M, Koyasu S. 1999. Interleukin 12-dependent interferon gamma production by CD8alpha+ lymphoid dendritic cells. J Exp Med 189:1981–1986.

[25] Oxenius A, Price DA, Eastderbrook PJ, O'Callaghan CA, Kelleher AD, Whelan JA, Sontag G, Sewell AK, Phillips RE. 2000. Early highly active antiretroviral therapy for acute HIV-1 infection preserves immune function of $CD8^+$ and $CD4^+$T lymphocytes. Proc Natl Acad Sci USA 97:3382–3387.

[26] Planz O, Ehl S, Furrer E, Horvath E, Brundler MA, Hengartner H, Zinkernagel RM. 1997. A critical role for neutralizing-antibody-producing B cells, CD4(+) T cells, and interferons in persistent and acute infections of mice with lymphocytic choriomeningitis virus: Implications for adoptive immunotherapy of virus carriers. Proc Natl Acad Sci USA 94:6874–6879.

[27] Rosenberg ES, Altfeld M, Poon SH, Phillips MN, Wilkes BM, Eldridge RL, Robbins GK, D'Aquila RT, Goulder PJ, Walker BD. 2000. Immune control of HIV-1 after early treatment of acute infection. Nature 407:523–526.

[28] Rosenberg ES, Billingsley JM, Caliendo AM, Boswell SL, Sax PE, Kalams SA, Walker BD. 1997. Vigorous HIV-1-specific $CD4^+$T cell responses associated with control of viremia. Science 278:1447–1450.

[29] Rossio JL, M T, Esser K, Suryanarayana DK, Schneider JW, Bess Jr, G M, Vasquez TA, Wiltrout E, Chertova MK, Grimes Q, Sattentau LO, Arthur LE, Henderson JD. 1998. Inactivation of human immunodeficiency virus type 1 infectivity with preservation of conformational and functional integrity of virion surface proteins. J Virol 72:7992–8001.

[30] Rowland-Jones S. 1999. HIV infection: Where have all the T cells gone? Lancet 354:5–7.

[31] Saha K, Bentsman G, Chess L, Volsky DJ. 1998. Endogenous production of beta-chemokines by $CD4^+$, but not $CD8^+$, T-cell clones correlates with the clinical state of human immunodeficiency virus type 1 (HIV-1)-infected individuals and may be responsible for blocking infection with non-syncytium-inducing HIV-1 *in vitro*. J Virol 72:876–881.

[32] Shedlock DJ, Shen H. 2003. Requirement for CD4 T cell help in generating functional CD8 T cell memory. Science 300:337–339.

[33] Subramani S, Mulligan R, Berg P. 1981. Expression of the mouse dihydrofolate reductase complementary deoxyribonucleic acid in simian virus 40 vectors. Mol Cell Biol 1:854–864.

[34] Sun JC, Bevan MJ. 2003. Defective CD8 T cell memory following acute infection without CD4 T cell help. Science 300:339–342.

[35] Van Coillie E, Van Damme J, Opdenakker G. 1999. The MCP/eotaxin subfamily of CC chemokines. Cytokine Growth Factor Rev 10:61–86.

[36] Verani A, Lusso P. 2002. Chemokines as natural HIV antagonists. Curr Mol Med 2:691–702.

[37] Wahren B, Morfeldt-Mansson L, Biberfeld G, Moberg L, Sonnerborg A, Ljungman P, Werner A, Kurth R, Gallo R, Bolognesi D. 1987. Characteristics of the specific cell-mediated immune response in human immunodeficiency virus infection. J Virol 61:2017–2023.

[38] Wilson JD, Imami N, Watkins A, Gill J, Hay P, Gazzard B, Westby M, Gotch FM. 2000. Loss of CD4+T cell proliferative ability but not loss of human immunodeficiency virus type 1 specificity equates with progression to disease. J Infect Dis 182:792–798.

[39] Yoshida A, Tanaka R, Murakami T, Takahashi Y, Koyanagi Y, Nakamura M, Ito M, Yamamoto N, Tanaka Y. 2003. Induction of protective immune responses against R5 human immunodeficiency virus type 1 (HIV-1) infection in hu-PBL-SCID mice by intrasplenic immunization with HIV-1-pulsed dendritic cells: Possible involvement of a novel factor of human CD4(+) T-cell origin. J Virol 77:8719–8728.

[40] Zhang L, Yu W, He T, Yu J, Caffrey RE, Dalmasso EA, Fu S, Pham T, Mei J, Ho JJ, Zhang W, Lopez P, Ho DD. 2002. Contribution of human $\alpha$-defensin 1, 2, and 3 to the anti-HIV-1 activity of CD8 anti-viral factor. Science 298:995–1000.

[41] Zhou P, Goldstein S, Devadas K, Tewari D, Notkins AL. 1997. Human CD4+ cells transfected with IL-16 cDNA are resistant to HIV-1 infection: Inhibition of mRNA expression. Nat Med 3:659–664.

